# Diagnostic value of the interferon-γ release assay for tuberculosis infection in patients with Behçet’s disease

**DOI:** 10.1186/s12879-019-3954-y

**Published:** 2019-04-15

**Authors:** Xiuhua Wu, Pang Chen, Wei Wei, Mengyu Zhou, Chaoran Li, Jinjing Liu, Lidan Zhao, Lifan Zhang, Yan Zhao, Xiaofeng Zeng, Xiaoqing Liu, Wenjie Zheng

**Affiliations:** 1 Department of Rheumatology and Clinical Immunology, Key Laboratory of Rheumatology and Clinical Immunology, Peking Union Medical College Hospital, Peking Union Medical College and Chinese Academy of Medical Sciences, Ministry of Education, Beijing, China; 2 Department of Rheumatology, Tianjin Medical University General Hospital, Tianjin, China; 3 Department of Rheumatology, Affiliated Mindong Hospital of Fujian Medical University, Fuan, Fujian China; 4 Department of Infectious Diseases, Clinical Epidemiology Unit, International Epidemiology Network, Centre for Tuberculosis Research, Chinese Academy of Medical Sciences and Peking Union Medical College Hospital, Beijing, China

**Keywords:** Behçet’s disease, Tuberculosis, Interferon-γ release assay, T-SPOT.TB

## Abstract

**Background:**

To investigate the diagnostic value of the interferon-γ release assay (IGRA) for detecting tuberculosis (TB) infection in patients with Behçet’s disease (BD).

**Methods:**

We retrospective analyzed the data collected from 173 BD patients hospitalized between 2010 and 2015. Ninety-nine healthy volunteers were enrolled as a control group. IGRA was performed using T-SPOT.TB. The diagnosis of active TB (ATB) was based on clinical, radiological, microbiological, histopathological information and the response to anti-TB therapy. Latent TB (LTB) infection was defined as asymptomatic patients with positive T-SPOT.TB.

**Results:**

TB infection was documented in 59 BD patients (34.1%). The sensitivity, specificity, positive predictive value, negative predictive value, positive likelihood ratio and negative likelihood ratio of T-SPOT.TB for the diagnosis of ATB were 88.9%, 74.8%, 29.1%, 98.3%, 3.53 and 0.15, respectively. The receiver-operating-characteristic curve demonstrated that spot-forming cells (SFCs) of 70/10^6^ PBMC was the optimal cutoff for diagnosing ATB, with an area under the curve of 0.891. Furthermore, the median SFCs in ATB group was significantly higher than those in LTB infection (466/10^6^ PBMC vs. 68/10^6^ PBMC, *p* = 0.007) or previous TB infection (466/10^6^ PBMC vs. 96/10^6^ PBMC, *p* = 0.018). A significant discrepancy between T-SPOT.TB and tuberculin skin test was noted (kappa coefficient = 0.391, *p* = 0.002).

**Conclusions:**

T-SPOT.TB, an IGRA, may assist in the diagnosis of ATB in BD patients, and the higher SFCs suggest ATB in BD patients.

## Background

Behcet’s disease (BD) is a multi-system inflammatory disorder, characterized by recurrent oral ulcers, genital ulcers, skin lesions, and ocular involvement of unknown etiology. Notably, the clinical manifestation of orogenital ulcers and skin lesions including erythema nodosum could be mimicked by tuberculosis (TB). Additionally, active TB (ATB) is frequently observed in patients with BD, and those symptoms were relieved after anti-TB therapy [1, 2]. BD and TB are also closely related in pathogenesis. *M.tuberculosis* may act as a trigger of BD through molecular mimicking. On the other hand, defective cell-mediated immunity in BD patients may increase the susceptibility of TB [3], and the use of glucocorticoids, immunosuppressants or biologic agents may increase the risk of reactivation of TB [4]. Therefore, it is crucial and challenging to identify TB in BD patients.

Tuberculin skin test (TST), a routine screening test for latent TB (LTB) infection, has limited diagnostic value in BD patients due to cross-reactivity with the bacillus Calmette-Guérin (BCG) vaccine and nontuberculous mycobacteria as well as pathergy reaction [5]. In additions, the autoimmune disorder or immunosuppressive status could lead to false-negative test results [6]. Interferon-γ release assays (IGRAs) are powerful assays for detection of TB [7], which detect the mycobacterial-antigen-specific interferon-γ-releasing T-cells in vitro*.* Given IGRAs do not cross-react with BCG vaccination or nontuberculous mycobacteria infection, IGRAs show higher specificity than TST [8]. IGRAs, including T-SPOT.TB, have been widely used to diagnose TB in autoimmune diseases, such as systemic lupus erythematosus (SLE) [8] and rheumatoid arthritis (RA) [9]. However, the diagnostic value of IGRAs for TB in BD patients remains unclear. To address this point, we retrospectively reviewed a cohort of BD patients tested with T-SPOT.TB and explored the diagnostic value of T-SPOT.TB for ATB in patients with BD.

## Methods

### Patients

Medical records of the hospitalized BD patients from Peking Union Medical College Hospital between January 2010 and March 2015 were retrospectively reviewed. All patients fulfilled the International Study Group BD criteria or the new International Criteria for BD [10, 11]. T-SPOT.TB was performed in BD patients when TB infection was suspected or corticosteroids and/or immunosuppressive drugs were planned to apply. We reviewed 268 BD patients with available medical records and confirmed 173 patients performed with T-SPOT.TB test. Of the 173 patients, clinical, laboratory and radiology data were collected and analyzed, and BD disease activity was assessed using the BD Current Activity Form 2006 (BDCAF2006). A total of 99 age- and sex-matched healthy volunteers were served as controls during the same period. T-SPOT.TB was performed in all participants. The diagnosis of ATB was based on clinical manifestations, radiologic findings, microbiology and histopathology, and the response to anti-TB therapy [12, 13]. ATB included culture-confirmed TB and the highly-probable TB. Culture-confirmed TB was defined as suggestive clinical and radiological findings and positive culture of *M.tuberculosis*. Highly-probable TB was defined as highly suggestive clinical and radiological features for TB in combination with the appropriate response to anti-TB treatment [12]. LTB infection was defined as asymptomatic patients with positive T.SPOT.TB [13].

### Interferon-γ release assays (IGRAs)

IGRAs were performed using T-SPOT.TB, a simplified enzyme-linked immunospot (ELISPOT) for detection of effector T cells that respond to stimulation by antigens specific for *M.tuberculosis*. Briefly, peripheral blood mononuclear cells were isolated from whole blood samples by density gradient separation, then stimulated with two TB specific antigens, early secreted antigenic target 6 (ESAT-6) and culture filtrate protein 10 (CFP-10), phytohaemagglutinin (PHA) (positive control) or AIM-V (GIBCOTMAIMV Medium liquid, Invitrogen, US) (negative control) in microplate wells (2.5 × 10^5^ per well) precoated with interferon-γ (IFN-γ) capture antibodies. The number of spot-forming cells (SFCs) was calculated after 16 to 20 h of incubation. The result was considered positive if the number of SFCs was six or more after subtracting the spot count of negative control [14].

### Tuberculin skin test (TST)

TST was performed with an intradermal injection using standardized 0.1 ml (5 U)-purified protein derivative into the ventral surface of the forearm according to the Mantoux method [15]. On the basis of National Institute for Health and Care Excellence (NICE) guidelines in 2016, the diameter of induration was measured in 48 to 72 h after administration 5 mm or more was defined as positive TST [16].

### Statistical analysis

Numerical data were expressed as mean ± standard deviation or median (interquartile range, IQR), and categorical data were expressed as frequencies/ percentages. The Mann-Whitney U test and Pearson chi-square test were used to compare differences between two groups. The concordance of TST and T-SPOT.TB results were assessed using κappa coefficient. (κappa > 0.75, excellent agreement; 0.40 to 0.75, fair to good agreement; and < 0.40, poor agreement). The diagnostic performance of T-SPOT.TB on ATB was evaluated by calculation of its sensitivity, specificity, positive predictive value (PPV), negative predictive value (NPV), positive likelihood ratio (PLR), and negative likelihood ratio (NLR). The area under the receiver operating characteristic curve of the T-SPOT.TB on peripheral blood mononuclear cell (PBMC) diagnostic cutoff for ATB was calculated. A two-sided with *p* values < 0.05 is considered statistically significant. Statistical analysis was performed by using SPSS (version 20.0, SPSS Inc., Chicago, IL, USA) and the GraphPad Prism (Version 5.01, GraphPad Software Inc., CA, USA).

## Results

### Characteristics of the study population

Among the 173 BD patients performed with TB-SPOT.TB test, 114 (65.9%) were men. The mean age was 37.07 ± 14.74 years, and median disease duration of BD was 84 months (range 1–608). The most common clinical manifestation was recurrent oral ulceration (98.3%). Other common findings included fever (81.5%), genital ulcers (58.4%), skin lesions (58.4%), gastrointestinal involvement (39.3%), vascular involvement (28.3%), positive pathergy test (22.5%), ocular involvement (19%), neurologic involvement (15%), cardiac involvement (9.8%) and hematological involvement (5.2%). The median BDCAF2006 score for disease activity was 2 (range 0–5). Elevated erythrocyte sedimentation rate (ESR) and high-sensitivity C-reactive protein (hs-CRP) levels were detected in 98 (56.6%) and 118 patients (68.2%), respectively. The median ESR was 21 (range 1–140) mm/first hour and the median hs-CRP was 11.54 (range 0.12–262.77) mg/L. In the past 2 years, 113 patients (65.3%) were treated with glucocorticoids, including 13 patients (7.5%) received glucocorticoid pulse therapy. Ninety-seven patients (56.1%) were under glucocorticoids treatment when T-SPOT.TB assay was performed, and the median daily dose of corticosteroids was 40 (range 5–100) mg. Ninety-four patients received immunosuppressants, including cyclophosphamide (30.1%), leflunomide (5.2%), cyclosporin A (4.6%), azathioprine (4.6%), methotrexate (4.6%) and salazosulfapyridine (2.9%). Sixteen patients (9.2%) received two or more immunosuppressants. Biological agents were used in 12 patients (6.9%), including infliximab injection in six patients, etanercept injection in five patients, etanercept followed by infliximab in one patient (Table 1).

**Table 1:** Demographics and Clinical characteristics of 173 patients with Behcet’s disease

Characteristics	BD (*n* = 173)	ATB (*n* = 18)	LTB (*n* = 29)	Previous TB (*n* = 12)	Non-TB (*n* = 114)
Age (years, mean ± SD)	37.07 ± 14.74	36.78 ± 10.61	43.52 ± 15.23	42.75 ± 14.06	34.87 ± 14.76
Male (percentage)	114 (65.9%)	11 (61.1%)	23 (79.3%)	5 (41.7%)	75 (65.8%)
BDCAF score (median, range)	2 (0–5)	1.5 (1–5)	2 (0–5)	3 (0–5)	2 (0–5)
Clinical features of BD					
Oral ulcer	170 (98.3%)	18 (100%)	28 (96.6%)	12 (100%)	112 (98.2%)
Genital ulcer	101 (58.4%)	15 (83.3%)	20 (68.9%)	9 (75%)	57 (50%)
Skin lesions	101 (58.4%)	**13 (72.2%)** ^**a**^	10 (34.5%)	10 (83.3%)	68 (59.6%)
Erythema nodosa	53 (30.6%)	**9 (50%)** ^**a**^	6 (20.6%)	5 (41.6%)	33 (28.9%)
Pathergy reaction	39 (22.5%)	7 (38.9%)	7 (24.1%)	3 (25%)	22 (19.3%)
Gastrointestinal involvement	68 (39.3%)	5 (27.7%)	12 (41.4%)	5 (41.7%)	46 (40.3%)
Vascular involvement	49 (28.3%)	7 (38.9%)	9 (31%)	3 (25%)	30 (26.3%)
Ocular involvement	33 (19%)	4 (22.2%)	3 (10.3%)	1 (8.3%)	25 (21.9%)
Neurologic involvement	26 (15%)	1 (5.5%)	3 (10.3%)	3 (25%)	19 (16.7%)
Treatment					
Glucocorticoid	113 (65.3%)	12 (66.7%)	22 (75.9%)	6 (50%)	73 (64%)
Immunosuppressive agents	94 (54.3%)	7 (38.9%)	16 (55.2%)	8 (66.7%)	63 (55.3%)
Biological agents					
Infliximab/Etanercept	12 (6.9%)	2 (11.1%)	2 (6.9%)	1 (8.3%)	7 (6.1%)

ATB compare with LTB; ^a^*p*<0.05

*BD* Behçet’s disease, *TB* tuberculosis, *ATB* active tuberculosis, *LTB* latent tuberculosis

### TB infection in BD patients

TB infection was documented in 59 BD patients (34.1%) in our study, including 18 (10.4%) ATB (BD-ATB), 29 (16.8%) LTB (BD-LTB) infection, and 12 (6.9%) patients with evidence of previous TB. Of the 18 BD-ATB cases, culture-confirmed TB and highly probable TB were diagnosed in 4 and 14 patients, respectively. Of the four culture-confirmed TB patients, pulmonary TB, tuberculous lymphadenitis, tuberculous pleuritis, tuberculous meningitis were diagnosed in one patient, respectively. Among 14 highly-probable TB, highly-probable pulmonary TB was diagnosed in 7 cases based on fever, cough, expectoration or chest pain, and CT-confirmed pulmonary cavity, infiltrating or nodules as well as *mediastinal lymphadenopathy*. Highly-probable tuberculous pericarditis was diagnosed in 2 cases presenting with fever and bloody pericardial effusion. Highly-probable tuberculosis of the knee joint was diagnosed in one case presenting with fever, swollen and painful knee, radiography showed the effusion, synovial thickening, and bone erosions in the knee. The other four patients had a history of TB, and presented with persistent fever, effusion or lymphadenopathy which are uncommon manifestations in BD. All cases with highly-probable TB had a good response to anti-TB treatment, but not corticosteroids and immunosuppressive drugs. Twenty-one patients were followed-up for 72.31 ± 16.45 months, and no ATB was observed. The clinical characteristics of different groups of BD patients were presented in Table 1. Interestingly, BD-ATB patients showed more tendency in developing skin lesions (72.2% vs 34.5%, *p* = 0.012), especially erythema nodosa (50% vs 20.6%, *p* = 0.036) compare to BD-LTB infection patients.

### The diagnostic value of T-SPOT.TB for ATB in BD patients

**Fig. 1:**
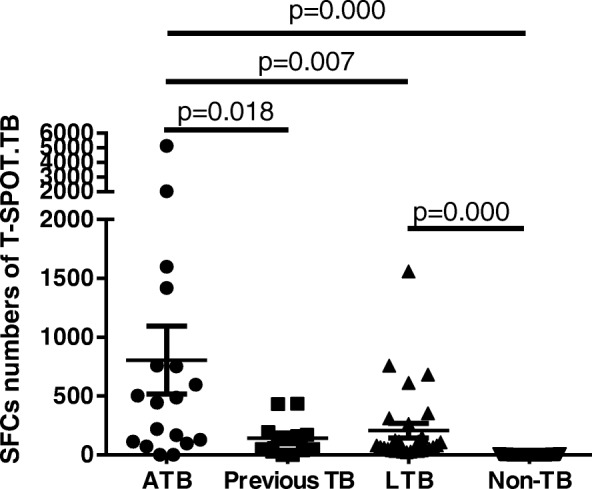
Comparison the number of SFCs between different groups of BD patients. The median SFCs of ATB(*n* = 18), previous TB (*n* = 12), LTB (*n* = 29), Non-TB(*n* = 114) was 466 (IQR: 108–925), 96 (IQR: 31.75–187), 68 (IQR: 38–208), and 2 (IQR: 0–3), respectively

Positive T-SPOT.TB tests were detected in 16 patients with ATB (88.9%), and the median number of spot-forming cells (SFCs) was 466/10^6^ PBMC (IQR: 108–925). Among patients with evidence of previous TB, ten patients (83.3%) had positive T-SPOT.TB with the median number of SFCs being 96/10^6^ PBMC (IQR: 31.75–187). The median number of SFCs in BD-LTB infection patients was 68/10^6^ PBMC (IQR: 38–208). The median number of SFCs in the BD-ATB group was higher than those in the BD-LTB infection group (*p* = 0.007) or those in previous TB patients (*p* = 0.018). The median numbers of SFCs in the BD-ATB group, BD-LTB infection group and previous TB patients were higher than those in the non-TB infection group (*p* = 0.000) (Fig. 1).

The sensitivity, specificity, PPV and NPV of the T-SPOT.TB test for the diagnosis of ATB were 88.9% (95%CI 63.9–98.1%), 74.8% (95%CI 67.1–81.3%), 29.1% (95%CI 18–43.1%), and 98.3% (95%CI 93.4–99.7%), respectively. PLR was 3.53(95%CI 2.57–4.85) and NLR was 0.15 (95%CI 0.04–0.55).

**Fig. 2:**
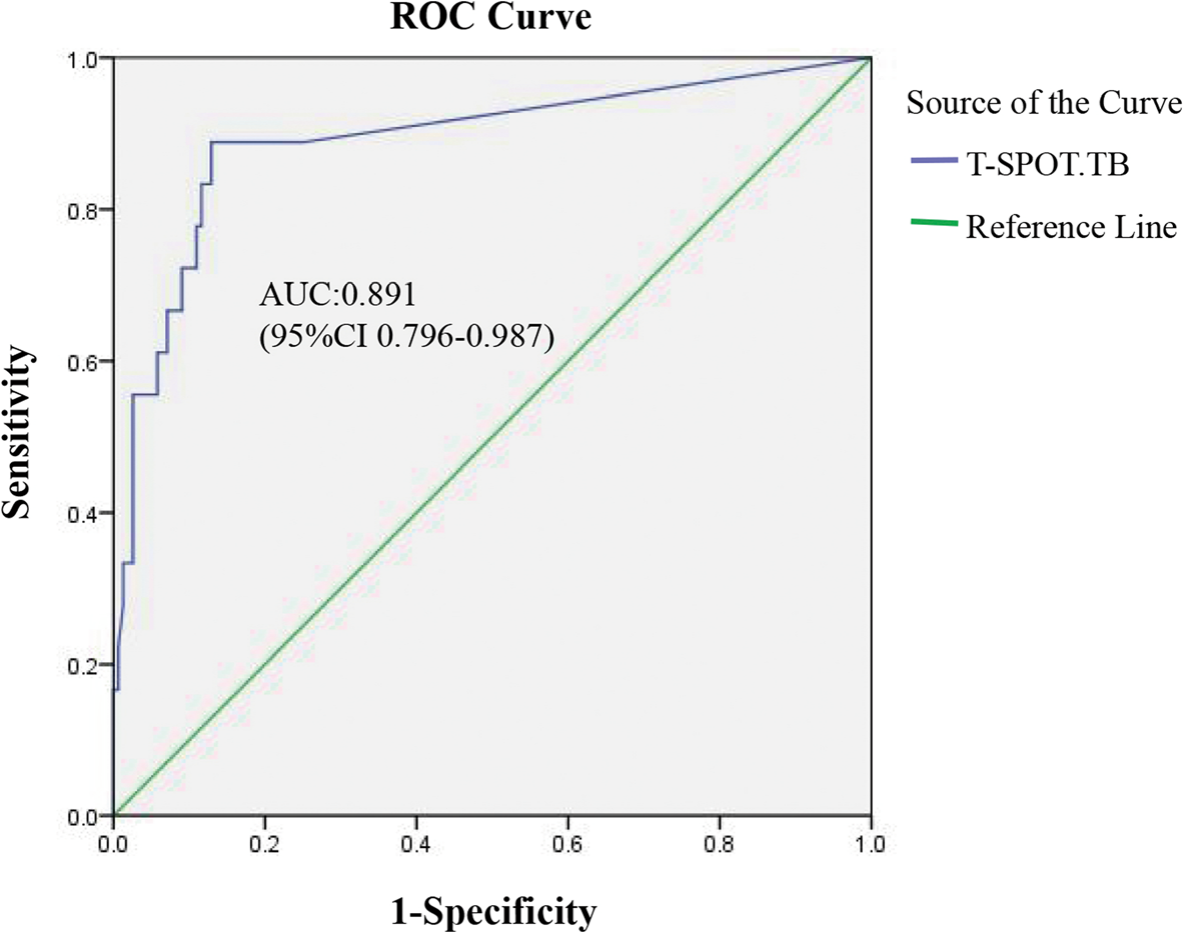
Receiver-operating-characteristic curve of T -SPOT.TB test in patients with Behcet’s disease

The receiver-operating-characteristic curve demonstrated that an SFCs of 70/10^6^ PBMC was the optimal cutoff for diagnosing BD-ATB, with an area under the curve of 0.891 (95%CI 0.796–0.987) (Fig. 2). Accordingly, the sensitivity, specificity, PPV, NPV, PLR and NLR was 88.9% (95%CI 63.9–98.1%), 87.1% (95%CI 80.5–91.8%), 44.4% (95%CI 28.3–61.7), 98.5% (95%CI 94.3–99.7%), 6.89 (95%CI 4.43–10.7), and 0.13 (95%CI 0.03–0.47), respectively.

LTB infection was documented in 27(27.3%) healthy controls, and the median number of SFCs was 192/10^6^ PBMC (IQR:80–308). There was no significant difference in the proportion of LTB infection (20.3% vs 27.3%, *p* = 0.205) and the number of SFCs detected by T-SPOT.TB (68/10^6^ PBMC vs 192/10^6^ PBMC, *p* = 0.079) between BD patients and healthy controls.

### The diagnostic values of T-SPOT.TB and TST for ATB

Both T-SPOT.TB and TST results were simultaneously tested in 63 patients. Among these patients, positive results of TST and T-SPOT.TB were presented in 44.4% (28/63) and 46% (29/63) patients, respectively. 30.2% (19/63) of the 63 patients had both positive TST and T-SPOT.TB, and 60.3% (38/63) had positive results on either TST or T-SPOT.TB (Table 2). A significant discrepancy between T-SPOT.TB and TST was noted (kappa coefficient of 0.391, 95% CI 0.146–0.617, *p* = 0.002).

**Table 2:** Discrepancy between T-SPOT.TB and TST (in 63 BD patients)

		T-SPOT.TB		
		Positive	Negative	Total
TST	Positive	19	9	28
	Negative	10	25	35
	Total	29	34	63

*TST* tuberculin skin test

The sensitivity of TST for detecting ATB was 78.6% (95%CI 48.8–94.3%), which was lower than that of the T-SPOT.TB test (92.9%), but there was no significant difference between the two groups. The specificity of TST was similar with T-SPOT.TB test (Table 3).

**Table 3:** Comparison the diagnostic values between T-SPOT.TB and TST and their combinations

	Sensitivity (%, 95CI)	Specificity (%, 95CI)	Positive predictive value (%, 95CI)	Negative predictive value (%, 95CI)	Positive likelihood ratio (95%CI)	Negative likelihood ratio (95%CI)
T-SPOT.TB	92.9 (64.2–99.6)	67.3 (52.3–79.6)	44.8 (27–64)	97 (82.5–99.8)	2.84 (1.85–4.36)	0.11 (0.0160.71)
TST	78.6 (48.8–94.3)	65.3 (50.2–77.9)	39.2 (22.1–59.3)	91.4 (75.8–97.8)	2.26 (1.41–3.63)	0.32 (0.12–0.91)
TSPOT.TB and TST (parallel)	92.9 (64.2–99.6)	48.9 (34.6–63.5)	34.2 (20.1–51.4)	96 (77.7–99.8)	1.82 (1.33–2.48)	0.15 (0.02–1.01)
TSPOT.TB and TST (serial)	78.5 (48.8–94.3)	83.7 (69.8–92.2)	57.8 (34–78.9)	93.2 (80.3–98.2)	4.81 (2.41–9.6)	0.26 (0.09–0.70)

*TST* tuberculin skin test

We also evaluated the diagnostic values of the combination of T-SPOT.TB and TST for ATB diagnosis in these 63 patients (Table 3) with parallel or serial manner, which was defined as either test was positive or both tests were positive, respectively. Serial test improved the specificity of diagnosis of ATB to 83.7% when combined T-SPOT.TB with TST, while no statistical significance was shown (67.3% vs 83.7%, *p* = 0.06). However, there was no difference in the sensitivity between parallel testing and T-SPOT.TB assay alone.

## Discussion

TB is an infectious disease primarily involving lungs and is endemic in developing countries. China has the world’s second largest TB prevalence, comprising around 9% of the world total according to the 2018 World Health Organization (WHO) TB report [17]. Patients with rheumatic diseases at high risk of TB infection, due to the immune dysregulation and the adverse effects of immunosuppressive agents [18–20]. *M.tuberculosis* culture, the gold standard of TB diagnosis usually takes 2–4 weeks. Therefore, a rapid diagnostic method of TB remains challenging. TST is a valuable screening test for TB infection [21], but the diagnostic value of TST in BD patients is compromised by the widely usage of the BCG vaccine in Chinese population and the immune dysfunction in BD patients [22].

In recent years, IGRAs emerges as accurate diagnostic assays for screening of TB infection. IGRAs specifically detect the presence of specific effector T cells which response to *M.tuberculosis*-specific antigens [23]. Dai Y. et al. systematically evaluated the diagnostic accuracy of T-SPOT.TB for detecting ATB in China, and found that the pooled sensitivity, specificity, PLR and NLR of T-SPOT.TB for the diagnosis of ATB were 88% (95%CI:86–91%), 89% (95%CI: 86–92%), 8.86 (95%CI: 5.42–14.46), and 0.13 (95% CI: 0.10–0.17), respectively [24]. And The ROC area under the curve (AUC) was 0.9548 (95% CI: 0.9323–0.9773) [24]. Consistently, Jiang B, et al. find that the sensitivity and specificity of T-SPOT.TB assays were 92.86 and 93.64%, in patients with rheumatic disease, which were higher than TST [25].

In this study, we evaluated T-SPOT.TB and TST for the diagnosis of ATB in BD patients. To our knowledge, this is the first study that reports the diagnostic value of T-SPOT.TB for TB in BD patients. We reported the high sensitivity (88.9%) and specificity (74.8%) of T-SPOT.TB for the diagnosis of ATB in BD patients. Compared to Dai Y’s study [24], our study showed similar results of sensitivity and NLR of the T-SPOT.TB test for the diagnosis of ATB while the lower specificity and PLR. The different study populations may explain this difference. Using SFCs> 70/10^6^ PBMC as the cutoff, the specificity and PLR are improved for diagnosing ATB in BD patients. Although, neither T-SPOT.TB nor TST can clearly distinguish between LTB infection, ATB or history of TB infection [23, 26], we found the median number of SFCs in BD-ATB group was higher than those in BD-LTB infection group and previous TB group, which suggested that higher SFCs in T-SPOT.TB test might indicate ATB. The numbers of IFN-γ producing T cells decreased during anti-tuberculous treatment, but IGRAs results remained positive at the end of treatment in most patients, which suggest a limited role of IGRAs in monitoring treatment efficacy [27]. Meanwhile, particular attention should be paid to ATB with negative IGRAs results, since those patients had poor outcomes, potentially due to delayed treatment [28].

It has been reported that the pooled sensitivity of T-SPOT.TB was higher than that of TST for the diagnosis of ATB in culture-confirmed or non-confirmed TB [29]. A discrepancy between T-SPOT.TB and TST test was noted in our study. The serial testing could increase the specificity of T-SPOT.TB for ATB diagnosis in BD patients when combined with TST, but the result didn’t reach statistical differences due to a limited number of cases. Further studies were required to explore the diagnostic efficiency between T-SPOT.TB and TST.

LTB infection is defined as the sustained immune response to *Mycobacterium tuberculosis* antigens with no evidence of clinically ATB. Given the risk of LTB infection reactivation increases in patients receiving immunosuppressive therapy or with immune dysfunction [30], it is important to identify LTB infection in BD patients. However, currently, there is no gold standard test for diagnosing LTB infection available. Several studies [31–33] suggested that IGRAs, including QuantiFERON-TB Gold In-Tube (QFT-GIT) and T-SPOT.TB, and TST, are all acceptable for screening LTB infection. In immune-mediated inflammatory diseases, the sensitivity and specificity of IRGAs in the diagnosis of LTB infection is higher than those in TST [34–36]. Also, T-SPOT.TB has been reported with higher sensitivity in detecting LTB infection than QFT-GIT and TST [32, 37], and Vassilopoulos et al. reported that the positive rates of QFT-GIT and T-SPOT.TB were 21 and 25%, respectively [38] . In our study, patients who had positive T -SPOT.TB but did not have ATB or history of TB infection were considered as LTB infection. The percentage of LTB infection in BD patients in our study was similar to that in other studies [32, 38]. A notable concern of IGRA in BD patients is the false positive or false negative rates of the IGRA test. Firstly, immunosuppressive agents or glucocorticoid might suppress T cell response to TB antigens and cause false negative IGRA test in BD patients. Nevertheless, a meta-analysis of IGRA in rheumatic patients showed that neither steroid nor disease-modifying anti-rheumatic drugs (DMARDs) significantly affect positive IGRA results [39]. Secondly, in BD patients with high disease activity, the systemic inflammation in BD potentially promotes T cell response via high-level proinflammatory cytokines. However, T cell response detected in IGRA is derived from the T cells with specific TCR recognizing tuberculosis-specific peptide, and systemic inflammation would not promote antigen-specific T cell response in the absence of specific peptide. Consistently, the SFCs of the negative control was less than 10 spots in our patients, which was comparable to those in healthy controls. Therefore, positive IGRA is strongly suggestive for latent TB in BD patients.

Our study has several limitations. First, our study is a retrospective analysis of a case series, which may have incomplete data of TST. Second, since all BD patients were enrolled from a single-center with relatively small sample sizes, and our center is a national referred center for complicated rheumatic diseases, potential selection bias of BD patients is possible. Third, it is challenging to identify active TB in BD patients based on clinical features since systemic inflammation is a shared feature of both BD and TB. The majority of our ATB patients were diagnosed according to clinical criteria rather than *M.tuberculosis* bacteriologically confirmed, which might introduce a high risk of bias. Fourth, given no gold standard of LTB infection is available, the precision of the diagnosis of LTB infection by T-SPOT.TB could not be assessed in this study. Nevertheless, this is the first study on the diagnostic value of T-SPOT.TB for TB in BD patients. A large-scale, multi-center study enrolled bacteriologically confirmed TB patients are warranted to confirm our findings.

## Conclusions

T-SPOT.TB may assist in the diagnosis of ATB in BD patients, and the higher number of SFCs (> 70/10^6^ PBMC) strongly suggested ATB. Further study is needed to investigate the diagnostic value of T-SPOT.TB for LTB infection in BD patients.

## Abbreviations

ATB: Active tuberculosis
BCG: Bacillus Calmette-Guérin
BD: Behcet’s disease
CFP-10: Culture filtrate protein 10
CRP: C-reactive protein
ELISPOT: Enzyme-linked immunospot
ESAT-6: Early secreted antigenic target 6
ESR: Erythrocyte sedimentation rate
IFN-γ: Interferon-γ
IGRAs: Interferon-γ release assays
IQR: Interquartile range
LTB: Latent tuberculosis
NICE: National Institute for Health and Care Excellence
NLR: Negative likelihood ratio
NPV: Negative predictive value
PBMC: Peripheral blood mononuclear cell
PHA: Phytohaemagglutinin
PLR: Positive likelihood ratio
PPV: Positive predictive value
QFT-GIT: QuantiFERON-TB Gold In-Tube
RA: Rheumatoid arthritis
SFCs: Spot-forming cells
SLE: Systemic lupus erythematosus
TB: Tuberculosis
TST: Tuberculin skin test
